# Pulmonary Complications in Connective Tissue Disease‐Associated Interstitial Lung Disease

**DOI:** 10.1111/crj.70116

**Published:** 2025-08-06

**Authors:** Agata Anna Lewandowska, Dorota Waśniowska, Cezary Rybacki, Michał Graczyk, Ola Duszyńska, Helena Mirus‐Arabik, Aleksandra Gaczkowska, Małgorzata Kołodziej

**Affiliations:** ^1^ Clinical Department of Pulmonology, Allergology and Pulmonary Oncology 10th Military Clinical Hospital With Polyclinic in Bydgoszcz Bydgoszcz Poland; ^2^ Faculty of Medicine Bydgoszcz University of Science and Technology Bydgoszcz Poland; ^3^ Department of Palliative Care, Collegium Medicum in Bydgoszcz Nicolaus Copernicus University in Toruń Poland; ^4^ Clinical Department of Oncology Oncology Center of Prof. Franciszek Lukaszczyk in Bydgoszcz Poland

**Keywords:** connective tissue disease, drug‐induced, exacerbation, infection, interstitial lung disease, rheumatic, tuberculosis

## Abstract

Interstitial lung disease (ILD) associated with connective tissue disease (CTD) is a challenging entity burdened with multiple risk factors and undesirable events. ILD can occur at any time and progress regardless of the underlying disease activity. Apart from the established difficulties in choosing an appropriate treatment strategy for both pulmonary and extrapulmonary involvement, such patients require a holistic and multidisciplinary approach. In an attempt to emphasize the significance of the problem and suggest potential research directions, the authors present four potentially most important and often misdiagnosed complications of the CTD‐associated ILD, in the form of acute exacerbation, drug‐induced pulmonary toxicity, nonspecific infection, and tuberculosis. Similar clinical manifestations of the patient's progressive deterioration, as well as the complex etiology of pulmonary involvement, raise controversies and force difficult therapeutic decisions. High morbidity and mortality among patients with progressive CTD‐associated ILDs necessitate further research in an attempt to enhance the treatment management in each CTD and improve the patients' quality of life.

## Graphical abstract

It is a challenge to distinguish and manage pulmonary complications associated with connective tissue disease‐associated interstitial lung disease, such as acute exacerbation, drug‐induced pulmonary toxicity, nonspecific infection and tuberculosis. Similar clinical manifestations and the diversity of radiological patterns raise controversies and force difficult therapeutic decisions.

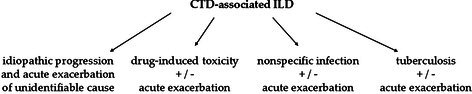

## 1 Introduction

Interstitial lung disease (ILD) is a collective term representing a heterogeneous group of parenchymal lung diseases characterized by varying degrees of inflammation and/or fibrosis, which result in impaired gas exchange and lung function [1, 2, 3]. ILDs are generally divided into idiopathic interstitial pneumonias, such as idiopathic pulmonary fibrosis (IPF), and diffuse parenchymal lung diseases, secondary to multiple environmental factors and systemic diseases [4, 5]. Lungs are abundant with collagen and blood vessels, making them susceptible to damage in the course of chronic connective tissue inflammation [6]. Therefore, the development of ILD is associated with several connective tissue diseases (CTDs), such as rheumatoid arthritis (RA), systemic sclerosis (SSc), poly‐/dermatomyositis (PM/DM), Sjögren's syndrome (SjS), systemic lupus erythematosus (SLE), as well as undifferentiated and mixed CTD [4, 5, 7, 8, 9].

**FIGURE 1:**
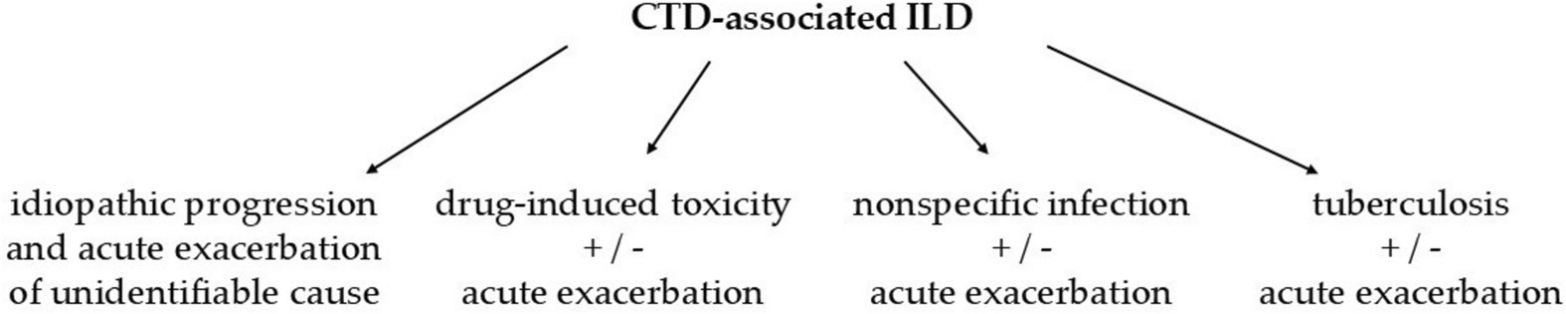
Pulmonary complications in CTD‐associated ILD.

CTD‐associated ILDs pose a challenge for clinicians regarding both diagnosis and treatment. Multiple confusing factors have to be considered, including the fact that the course of ILD does not always follow the activity of the underlying CTD [10, 11, 12, 13]. Deterioration of the patient's condition requires a multidisciplinary and holistic approach in order to understand its underlying cause and enable appropriate therapeutic decisions. The purpose of this review is to present four potentially most important complications posed to the patients with CTD‐associated ILDs, such as acute exacerbation (AE), drug‐induced toxicity, nonspecific infection, and tuberculosis (Figure 1). The aim is to highlight the dilemmas encountered in clinical practice and identify potential research areas, which could enhance the treatment and prognosis of these patients in the future.

The review was based on literature available in PubMed, Scopus, and Web of Science medical databases. A thematic analysis was conducted on relevant articles dated between 2000 and 2025; however, the tuberculosis section includes several references from before 2000 due to limited literature on the subject published in the 21st century. The keywords used to perform the search included the following: “interstitial lung disease”, “connective tissue disease”, “rheumatic”, “acute exacerbation”, “drug‐induced toxicity”, “infection”, and “tuberculosis”. The review encompassed review articles, meta‐analyses, systematic reviews, case series, society guidelines, as well as prospective and retrospective studies. The search was restricted to articles published in English.

## 2 Connective Tissue Diseases Associated With ILD

**FIGURE 2:**
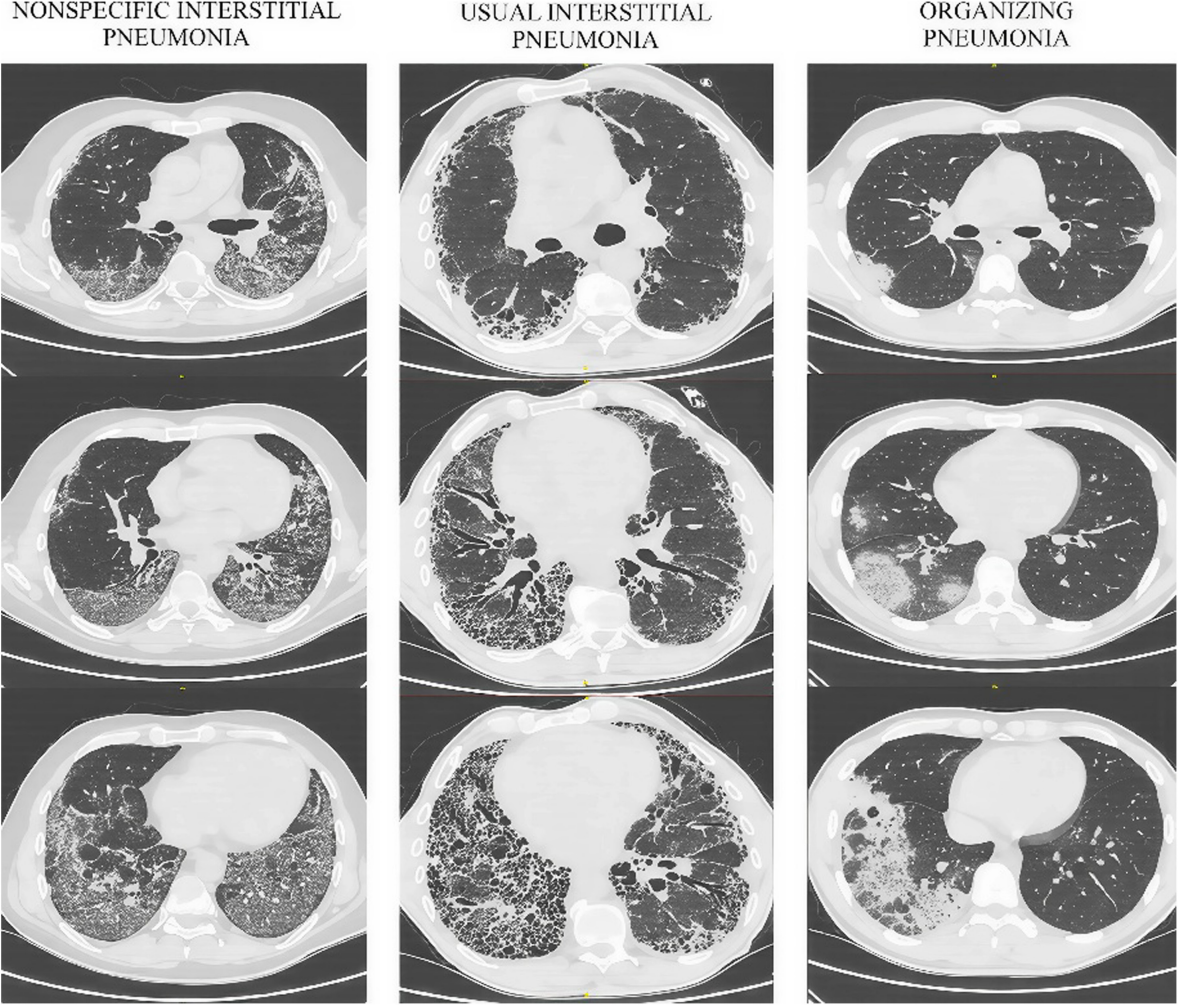
Common radiographic signs and patterns in interstitial lung disease visualized in HRCT.

The lesions visualized in high‐resolution computed tomography (HRCT) are dominated by inflammation, fibrosis, or the combination of both [4]. Depending on the radiological and histological examination, there are several distinguished patterns of ILDs, such as nonspecific interstitial pneumonia (NSIP), usual interstitial pneumonia (UIP), organizing pneumonia (OP), diffuse alveolar damage (DAD), desquamative interstitial pneumonia, acute interstitial pneumonia, or lymphocytic interstitial pneumonia [4, 5]. Although each CTD demonstrates a different pattern of parenchymal involvement, certain similarities can be observed [7]. The NSIP pattern, associated with reticular opacities in subpleural and basal parts of the lungs, is considered the most common [7]. Different patterns of ILD with their characteristic radiological findings are presented in Table 1 and Figure 2. Advanced, chronic lesions are associated with fibrosis in the form of reticulation, traction bronchiectasis, and honeycombing [7].

**TABLE 1:** Common histological and radiological patterns in CTD‐associated ILDs [4, 5, 7, 14].

Nonspecific interstitial pneumonia	Usual interstitial pneumonia	Organizing pneumonia	Diffuse alveolar damage	Lymphocytic interstitial pneumonia
Bilateral basilar reticular markings and ground glass opacities	Peripheral and basilar reticulonodular opacities with reticulation, honeycombing and traction bronchiectasis	Patchy airspace consolidations with ground glass opacification	Bilateral consolidation, diffuse ground glass opacities	Thin‐walled cysts with ground glass opacities and centrilobular nodules

It is estimated that up to 58% of patients with RA—the most common CTD—can be affected by lung disease [5, 13, 15]. The clinical manifestation usually includes dry cough, progressive dyspnea, and a restrictive ventilatory defect with reduced diffusion capacity of carbon monoxide on pulmonary function testing [7, 13]. Dyspnea on exertion is usually not conspicuous among patients with RA, who do not engage in intensive physical activity due to arthritic symptoms [13]. However, in up to 20% of cases, pulmonary manifestation can precede articular symptoms [8]. Patients can remain asymptomatic for a long time until a significant progression occurs [13]. RA‐associated ILD most often manifests as the UIP pattern, less commonly—the NSIP pattern [7, 16].

SSc is a connective tissue disease most often associated with ILD (up to 91% of patients), usually in the form of the NSIP‐pattern injury, less commonly the UIP‐pattern [4, 7, 17, 18]. Together with pulmonary hypertension, they constitute the major causes of death in patients with SSc [4, 7].

In PM/DM, pulmonary involvement in the form of combined NSIP and OP patterns, or less commonly the UIP‐pattern, is observed in up to 80% of patients, and constitutes a common cause of mortality [7, 19]. The subacute onset of dyspnea and extensive basilar ground glass opacities with reticular interstitial pattern indicate the more aggressive type of ILD, with a tendency to progress over weeks or months, and resist treatment [7].

In SjS, pulmonary involvement is estimated to be 10%–27% [8]; however, a clinically relevant ILD is less common and usually takes a self‐limiting course [7]. It is worth mentioning that SjS can be easily missed in patients with the UIP‐pattern and diagnosed as IPF or unclassifiable ILD if the ILD precedes the sicca syndrome and the patients are seronegative at the time of diagnosis [8]. Additionally, impaired immune function of the mucosal barrier predisposes those patients to respiratory tract infections [7].

In SLE, mild pulmonary involvement, usually in the form of NSIP‐pattern, is observed in approximately one‐third of patients; however, clinical relevance applies only to 2%–13% [4, 5, 7, 8]. The acute onset of symptoms (dyspnea, fever, cough, hemoptysis) with diffuse, bilateral ground glass opacities visualized in HRCT indicate acute lupus pneumonitis and possibly diffuse alveolar hemorrhage—severe conditions with high mortality, often requiring mechanical ventilation [7].

A large number of patients with ILD present positive serologies and extrapulmonary symptoms indicating the presence of an underlying autoimmune disease; however, they do not meet the diagnostic criteria for any specific CTD [7]. In such cases, they are classified into undifferentiated CTD [7].

In most CTDs, lung involvement can occur at any time in the course of the disease, regardless of the progression of extrapulmonary lesions [6, 7]. The reported prevalence of ILD in all CTDs varies significantly among studies, which necessitates further investigation [8]. The need for periodic follow‐up is justified, as the treatment of asymptomatic patients with normal lung function is considered unnecessary [5]. The prognosis worsens significantly in the presence of pulmonary fibrosis, whereas centrilobular nodules indicate a lower risk of progression [6]. Further research is required in order to assess the risk of ILD progression in each CTD [5].

Safe and effective therapies for CTD‐associated ILDs are still lacking [4, 8, 20, 21]. The therapy is based on disease‐modifying antirheumatic drugs, glucocorticosteroids (GCS) and/or antifibrotic agents (nintedanib) [5, 6, 8, 10, 22, 23, 24]. Despite achieving a satisfactory response to immunosuppressives in the treatment of rheumatologic diseases, the efficacy of these drugs in ILDs remains moderate [4, 5]. A detailed discussion regarding the treatment in each CTD is beyond the scope of this review. Considering the significant morbidity and mortality, further research on CTD‐associated ILD is necessary in an attempt to understand its pathogenesis better and enhance the treatment management in each entity [4, 5, 6, 7, 21].

Adequate diagnosis largely depends on the cooperation of pulmonologists and rheumatologists [4, 25]. The interdisciplinary approach and joint consultations are valuable for determining personalized treatment; however, they are often hard to coordinate [25]. Additionally, pulmonologists provide beneficial input regarding the potential complications associated with the disease progression, such as respiratory failure, the need for antifibrotic therapy, lung transplantation, and palliative care [25].

## 3 Interstitial Pneumonia With Autoimmune Features

Approximately 14% of patients with ILD manifest autoimmune features, but do not fulfill the diagnostic criteria for a definite CTD [26, 27]. To address the issue, a research entity of interstitial pneumonia with autoimmune features (IPAF) was proposed in 2015 as an overlap between idiopathic interstitial pneumonia, such as IPF, and CTD‐associated ILDs [26, 28, 29]. The current consensus definition includes radiological or histopathological evidence of interstitial pneumonia (such as NSIP, OP, NSIP with OP overlap and LIP), exclusion of alternative etiologies for interstitial pneumonia, as well as incomplete features of a defined CTD [26]. The prevalence of IPAF is unknown and varies between 7% and 34% of all ILDs depending on the region [26, 27]; however, the decision on how far to search for the evidence of an underlying CTD often determines the final diagnosis [27]. Although patients with IPAF are additionally at risk of progression to a defined rheumatic disease [27], their prognosis is better than for patients with idiopathic ILD, with an average 5‐year survival of 70% [30].

Considering that IPAF is a research entity rather than a diagnosis, it is uncertain whether the management should be extrapolated more from IPF or CTD‐ILD treatment strategies [27, 28, 31]. As the literature regarding IPAF management is limited and there are no randomized controlled trials targeting it specifically, further research is necessary to determine the optimal therapy in this group of patients [27, 28]. Possible treatment options include observation without drug therapy, immunomodulation with GCS and/or immunosuppressants, as well as antifibrotic agents [27, 28]. Considering the significant heterogeneity within the patients with IPAF, therapeutic decisions majorly rely on an individual risk–benefit assessment, preferably discussed in a multidisciplinary setting [26].

## 4 Acute Exacerbations of CTD‐Associated ILDs

An exacerbation of ILD is generally defined as a rapid (within 1 month) worsening of dyspnea with documented new bilateral ground glass opacity and/or consolidations superimposed on ILD‐consistent pattern visualized in HRCT, together with the exclusion of other causes, such as pulmonary embolism, pneumothorax, fluid overload, or heart failure [9, 32, 33, 34]. The last condition is particularly important as patients with CTD‐associated ILD have a 1.65 times higher risk of cardiovascular disease and increased prevalence of heart failure and arrhythmia [35].

Exacerbations of CTD‐associated ILDs are increasingly recognized in clinical practice and burdened with a devastating disease course and high mortality [5, 11, 22, 33, 34, 36]. They seem to be linked to the severity of the underlying ILD and the presence of the UIP pattern [37]. The initial reports evaluated the incidence of AEs in CTD‐associated ILDs to be 3.19 and 5.77/100 patients/year, which is lower compared to the number of AEs among patients with IPF [11, 22]. Another study estimated the 90‐day all‐cause mortality rate in patients with exacerbated CTD‐associated ILD to vary between 30% and 58.3% [22]. However, larger studies are necessary as the available literature still does not reflect the clinical significance of AEs in CTD‐associated ILDs, which varies in each rheumatic disease [11].

It is unfortunate that the diagnosis of AE is based almost exclusively on radiological findings and unspecific clinical presentation, mainly in the form of rapid respiratory decompensation [33]. Generally, the diagnostic criteria acknowledge potential triggers provoking AE, such as infection, microaspiration, gastroesophageal reflux, surgical procedures, air pollution, or progression of the primary interstitial process [34]. However, a specific definition of AE secondary to CTD has not been standardized yet [37]. It could be defined as an acute, clinically significant respiratory deterioration characterized by new widespread alveolar abnormalities (as in all ILD types), additionally highlighting characteristic triggers of AE in CTD‐associated ILD in the form of antirheumatic treatment and its complications [37, 38]. These unique aspects distinguish the definition from exacerbations in patients with IPF [37]. It has not been confirmed whether a triggered exacerbation in the course of CTD‐associated ILD has a worse outcome than an idiopathic one, related to the intrinsic acceleration of the disease [34, 39]. Nevertheless, most patients surviving AE experience impaired quality of life and poor long‐term prognosis [34, 40]. Due to the lack of reliable biomarkers for detecting and monitoring the progression of the disease, several serum cytokines were examined for their clinical utility [9, 41, 42]. Elevated levels of IL‐6, IL‐8, and TNFα were associated with the occurrence of AE in CTD‐associated ILD [41]. Moreover, the combination of three variables, such as elevated IL‐6 levels, total bilirubin, and decreased CD3 + CD4 + T cell counts, predicted the AE better than any single biomarker [41].

The definition, diagnostic criteria, and treatment strategy of AE are based mainly on the management indicated for patients with IPF [34]. The clinical problem is of particular importance as AE in the course of CTD‐associated ILD requires advanced therapeutic management with the intensification of immunosuppressive therapy, which has not been standardized yet [20, 33, 34, 41, 43]. High‐dose GCS, cyclophosphamide, and rituximab have been proposed in AEs of CTD‐associated ILD; however, the existing evidence remains limited [34]. Doses of GCS are variable and can include pulse dosing of methylprednisolone (500–1000 mg per day) or lower initial doses of prednisone (1 mg/kg per day), depending on the severity of the exacerbation [37, 38]. It is accepted to administer GCS after collecting bacterial cultures and starting broad‐spectrum antibiotic therapy [37]. Oxygen supplementation in the form of high‐flow nasal cannula and non‐invasive positive pressure ventilation is indicated, while more aggressive therapies, such as mechanical ventilation and extracorporeal membrane oxygenation, should generally be restricted to potential transplant candidates [37, 38, 44]. The decision to intubate should be preceded by a discussion with the patient and their caregivers to ensure the understanding of realistic therapeutic goals [37]. Early implementation of palliative care and regular counseling is advisable to improve the patient's quality of life throughout the disease course [37]. In terminally ill patients with poor short‐term prognosis, opioids are an effective treatment strategy for dyspnea [37].

## 5 Drug‐Induced Toxicity

A large number of anti‐inflammatory, antimicrobial, as well as biologic and nonbiologic antirheumatic drugs are reported to cause pulmonary complications, including interstitial pneumonia, fibrosis, bronchospasm, pulmonary edema, and pleural effusion [6, 14]. Drug‐induced pulmonary toxicity, including AEs of the pre‐existing ILD, is difficult to establish, generally underdiagnosed, and often identified only by exclusion of other possible causes of clinical deterioration, such as infection, tuberculosis, congestive heart failure, or cancerous lymphangitis [6, 14, 45, 46].

According to collectable data, the incidence of drug‐induced lung disease varies between 4.1 and 12.4 patients/million/year and accounts for 3%–5% of ILD cases [47]. Older studies indicated that methotrexate, leflunomide, tacrolimus, gold salts, sulfasalazine, as well as anti‐tumor necrosis factor α (anti‐TNFα) and anti‐CD20 biologic agents exhibited the highest risk of ILD development [6, 7, 13, 22]. Due to concerns about the pulmonary toxicity of methotrexate and TNF inhibitors, alternative drugs were considered, such as abatacept, IL‐6 inhibitors (tocilizumab, sarilumab), rituximab, and Janus kinase inhibitors (tofacitinib, baricitinib, upadacitinib) [48]. No significant differences in mortality or respiratory hospitalization were found, which emphasizes the need for research on advanced immunomodulatory therapies [48]. However, new data suggest that the incidence of AE in RA‐associated ILD among GCS and DMARD users (especially methotrexate and TNFα inhibitors) is not as high as previously thought [49, 50]. Additionally, a meta‐analysis of data from 15 studies on overall mortality showed that methotrexate significantly reduced mortality in patients with RA‐associated ILD [50, 51]. Methotrexate, as well as tofacitinib, may even have a protective role in RA‐associated ILD risk [52]. Although the influence of DMARDs on ILD development and aggravation is still a subject of controversy, the results of recent meta‐analyses partially alleviated the concerns regarding their use in patients with RA [50, 52].

There is no characteristic pattern of drug‐induced lung disease; however, the most common ones include NSIP, OP, DAD, simple pulmonary eosinophilia, and hypersensitivity pneumonia [6, 14, 53]. The onset and/or exacerbation of drug‐induced ILD are largely unpredictable; however, there are identified risk factors that include underlying conditions associated with ILD, such as connective tissue disorders, as well as dose‐dependent toxicity and genetic predisposition [53]. Patients with drug‐induced pneumonitis present unspecific symptoms in the form of dyspnea, fever, and deteriorated exercise tolerance [14]. The clinical image varies from mild to rapidly progressive and is associated with high mortality [53]. The drug‐induced ILD can develop within the first few days of the drug administration or after several years [45]. The connection is hard to objectify, especially in the case of multidrug therapy [14]. The diagnosis follows several criteria, such as the history of drug exposure consistent with clinical, radiological, and histopathological images associated with the causative agent, improvement after the agent withdrawal, and exclusion of other causes of ILD [14].

The prognosis is uncertain and depends on several factors, such as the drug itself, the patient's comorbidities, and the severity of lung disease [14]. Acute episodes usually resolve within 24–48 h after the drug discontinuation; however, chronic types may require a longer period of time [14]. In some cases, the disease does not improve or progresses continuously even after the drug withdrawal [14]. GCS are used in the progressive type of the disease; however, it is the permanent discontinuation of the causative agent that constitutes the cornerstone of the treatment [6, 53]. Considering there are no consistent recommendations regarding the drug‐induced ILD management or convincing evidence from meta‐analyses for the use of GCS, prospective studies are required [47].

The question remains whether the patient's deterioration is related to the progression of the pre‐existing CTD‐associated ILD, an adverse drug reaction, or possibly both—coexisting and prompting each other [6]. In the case of delayed response and further progression after the drug withdrawal, the differential diagnosis becomes extremely difficult [7, 45]. The usage of drugs associated with high rates of pulmonary complications in patients with pre‐existing lung disease is controversial and undoubtedly a difficult therapeutic decision [7]. Apart from GCS, relatively safer treatment options for CTDs include cyclophosphamide, azathioprine, cyclosporine, mycophenolate mofetil, as well as certain biologic agents, which, despite their extrapulmonary efficacy, show modest effect on ILD [7]. A large number of potentially toxic drugs, together with the lack of definitive guidelines and randomized clinical trials, forces the treatment to be conducted mostly empirically [7].

## 6 Nonspecific Infection

Viral, bacterial, and fungal infections are potentially involved in the pathogenesis of ILDs, and as triggers of AEs [54, 55]. Increased risk of infection is associated with age, diabetes, dosage of steroids, hypoalbuminemia, and others [56]. Infections are considered the major cause of death in several CTD‐associated ILDs, partially due to the drug‐induced immunosuppression and leukopenia [13]. Several small retrospective and postmortem studies indicate that infections are detected in up to 30% of patients with AE and acute respiratory decline [55].

The patient's immune function status should always be taken into consideration [6]. The significantly increased risk of bacterial infection in patients with a suspected AE of a CTD‐associated ILD highlights the critical importance of ruling the infection out first, before starting aggressive immunosuppressive treatment [6, 7]. In the case of a suspected acute respiratory tract infection, additional collection of sputum and bronchial aspirate for non‐specific flora and tuberculosis (TB) cultures may be necessary [13]. However, according to one study, there are no differences in the outcomes for patients with AE of fibrotic lung disease compared to those additionally suffering from infection [57]. So far, no association has been found between AE and any specific microorganism [58]. It is worth mentioning that infections with various pathogens, such as *Mycobacterium tuberculosis* , *Mycobacterium avium, Pneumocystis jirovecii*, and fungi can cause radiological lung lesions similar to those visualized in the course of CTD‐associated ILDs, and thus mislead clinicians [13].

Procalcitonin is useful in detecting bacterial origins and monitoring the efficacy of antibiotic therapy [32]. High probability of acute infection usually prompts the decision for early administration of antibiotics and reduction of immunosuppression [58]. On the other hand, intensification of the immunosuppressive therapy is crucial for disease control in the management of idiopathic AE [58].

Patients with ILD are also susceptible to chronic respiratory tract infections, which facilitate disease progression and impede the diagnosis due to pre‐existing fibrotic lesions [13, 58]. Perhaps, targeted therapy for chronic infections, as well as prophylactic antibiotics in case of recurrent infections should be considered [58]. On the other hand, the use of prolonged antibiotic therapy in patients without concomitant bacterial infection is associated with an increased risk for fungal infections and antibiotic resistance development [32].

## 7 Tuberculosis

TB is a disease caused by *M. tuberculosis* —an intracellular pathogenic bacterium characterized by the ability to influence the human immune system by unique antibacterial mechanisms and remain within the host for years [59]. It is classified in the *M. tuberculosis* complex group, which includes several other pathogenic mycobacteria species, such as *M. canettii, M. microti, M. bovis*, and *M. africanum* [60, 61].

Approximately 1.7–2 billion people worldwide have been infected with *M. tuberculosis* and 1.2–1.5 million per year die from the disease, with the most cases reported in the areas of South‐East Asia, Africa, and the Western Pacific [59, 62, 63]. Latent *M. tuberculosis* infection (LTBI) is a persistent immune reaction to the stimulation of *M. tuberculosis* antigens, which cannot be classified as an active disease [64]. According to the World Health Organization (WHO), such a condition pertains to one‐fourth of the population in the world [64]. The prevalence of LTBI in patients with rheumatic diseases differs worldwide, with 20.4% in India and 29.5% in Brazil [65, 66]. Only 5 to 10% of the population affected by LTBI will ever progress to the active state of the disease; however, the risk increases substantially in immunocompromised patients [64].

Reports indicate that the incidence of *M. tuberculosis* infections in patients with ILD is estimated to be four to five times higher than that of the general population [67, 68, 69]. Interestingly, *Mycobacterium* is more common not only in patients with IPF treated with immunosuppressants, but also in those who did not receive such treatment [67]. As extensive lung tissue destruction potentially increases the susceptibility to TB development, and ILD exacerbations perhaps contribute to it as well [70, 71]. The processes of TB development in patients with ILD are still poorly understood [70].

Tumor necrosis factor (TNF) α is an important pro‐inflammatory cytokine produced by macrophages and monocytes in the course of mycobacterial infection [64, 72]. Its release induces phagocytosis and granuloma formation, therefore preventing pathogen proliferation [64]. The treatment of various rheumatic diseases involving anti‐TNF biologic agents and other immunosuppressives provides numerous benefits by reducing inflammation and inhibiting progressive tissue damage associated with chronic disorders [64]. However, due to their mechanism of action, the administration of these agents is associated with the risk of active TB development and the necessity to monitor the patients annually during the treatment [64, 73, 74, 75, 76, 77]. The infection most often affects the lungs; however, it can also involve skin, nervous system, eyes, lymph nodes, joints and bones, genitourinary, and the abdomen [59].

Primary pulmonary TB may involve lung parenchyma, lymph nodes, tracheobronchial tree, and pleura [78, 79]. Four entities are usually distinguished, such as gangliopulmonary TB—characteristic of primary TB, TB pleuritis, miliary TB, and tracheobronchial TB [80, 81]. The primary disease is usually self‐limiting and asymptomatic in immunocompetent individuals, and its detection occurs retrospectively by the detection of calcified hilar or paratracheal lymph nodes and parenchymal scarring in radiological imaging [78].

Post‐primary TB in about 90% of cases occurs in immunocompromised and malnourished individuals as a result of reactivation of dormant bacilli, or less frequently, in the course of reinfection [82]. Post‐primary pulmonary TB involves liquefaction of caseous necrosis with subsequent cavity formation, progressive fibrosis, and parenchyma destruction, bronchogenic spread, and pleural involvement (more common compared to primary TB) [78, 83, 84, 85]. Apicoposterior segments of the upper lobes and superior segments of the lower lobes are typical areas of involvement [78].

Tuberculin skin test or interferon γ (INFγ) release assay is used as a proxy for LTBI [86]. In immunocompromised patients and/or receiving GCS therapy, both tests can present falsely negative results [86, 87]. Apart from the radiological imaging, sputum smear microscopy and culture constitute the initial tests performed in case of suspected TB [78]. However, negative results of induced‐sputum samples do not certainly rule out active tuberculosis [88]. Strong suspicion of lower respiratory tract infection or active pulmonary TB prompts the decision to conduct invasive examination in the form of bronchial aspirate collection at bronchoscopy [88, 89, 90, 91]. Unfortunately, the availability of bronchoscopy is often limited due to costs and logistical challenges [88].

**FIGURE 3:**
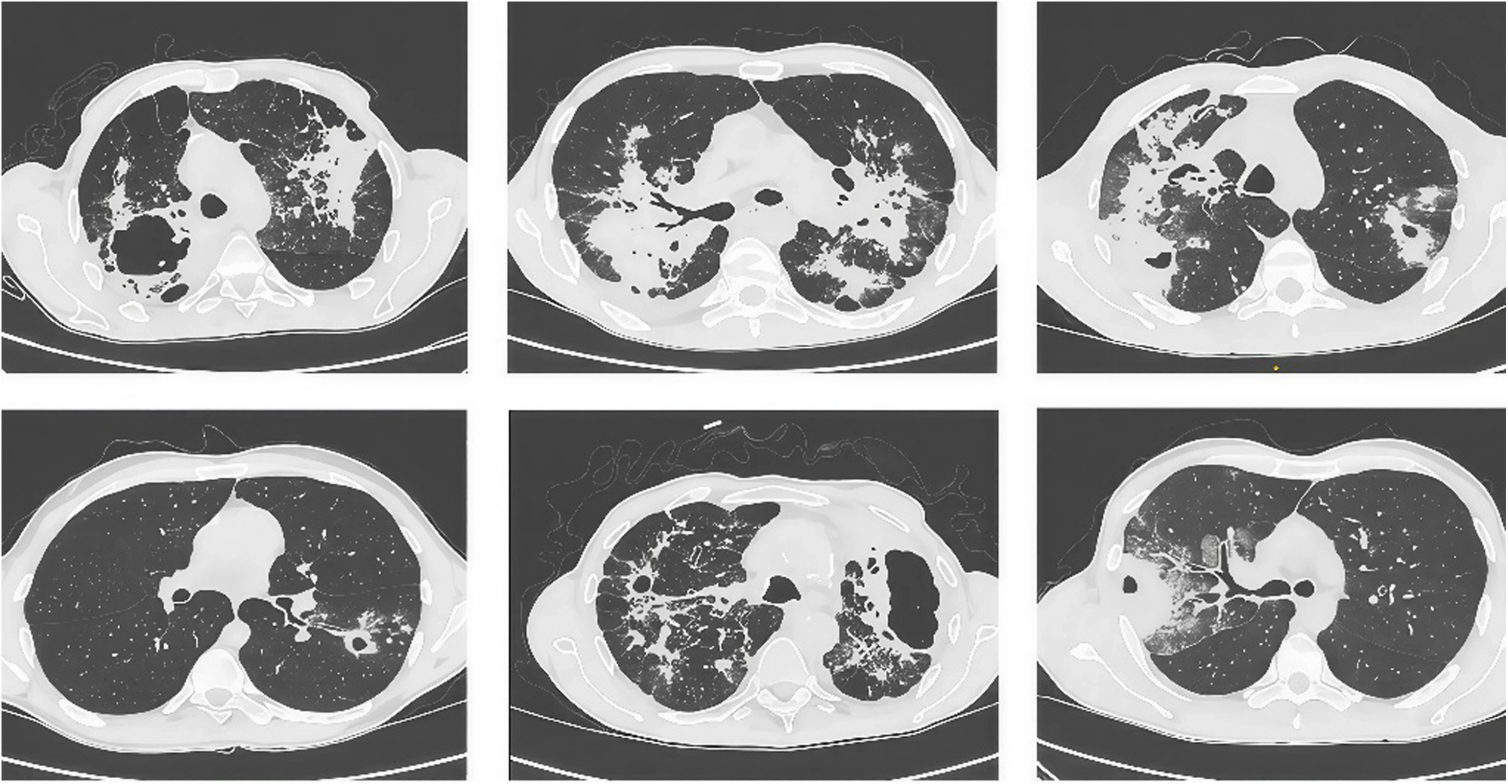
Examples of radiological findings in active tuberculosis visualized in HRCT.

In active TB, computed tomography scans commonly show centrilobular nodules, “tree‐in‐bud” branching opacities and thick‐walled cavities with surrounding consolidations [92, 93]. Characteristic radiological findings indicating active primary and post‐primary TB are presented in Table 2 and Figure 3. Thin‐walled cavities, tuberculomas, fibrosis, isolated pleural thickening, and calcification suggest a history of healing [78]. It is worth noting that cavitary lesions should be differentiated from bullae, cysts, or pneumatoceles [78]. Tree‐in‐bud nodules can also be observed in bacterial, fungal, viral, and parasitic infections, as well as in the presence of bronchiolitis, connective tissue disorders, or substance aspiration [78]. Air‐fluid levels in the cavities indicate concomitant bacterial or fungal infection associated with abscess formation, or possibly empyema, with bronchopleural fistula and pleural effusion subsequent to the cavity rupture [94, 95]. In the case of pleural effusion, thoracocentesis with fluid analysis should be performed [78, 85].

**TABLE 2:** Characteristic radiological findings indicating active primary and post‐primary tuberculosis [78].

Primary tuberculosis	Ipsilateral hilar and paratracheal lymph nodes enlargement
	Enlarged mediastinal lymph nodes with central necrosis and heterogenous enhancement
	Diffusely spread miliary nodules
	Involvement of apicoposterior segments of the upper lobes and superior segments of the lower lobes
Post‐primary tuberculosis	Centrilobular nodules with “tree‐in‐bud” branching opacities and bronchial wall thickening
	Progressive consolidations and infiltrations with the destruction of lung parenchyma
	Peribronchial clusters of nodular opacity
	Regions of caseous necrosis and formation of thick‐walled cavities with surrounding consolidations
	Unilateral pleural effusion/empyema

The diagnosis of active TB in patients with ILD remains a challenge as it may successfully mimic other diseases [96]. New consolidations and opacities can both be the result of AE, TB reactivation, or other superimposed infections [78]. Pre‐existing fibrotic lesions mask new consolidations and further complicate the diagnosis [97, 98, 99]. Atypical computed tomography findings in a small group of patients with IPF with confirmed TB included subpleural nodules and lobar/segmental consolidations located most commonly in the right lower lobe [96]. Possible absence of the typical radiological image of active TB in patients with ILD can result in prolonged and unnecessary antibiotic therapy [70].

As LTBI treatment strategies differ worldwide, local guidelines and expert opinion should be considered and followed [100]. Isoniazid in monotherapy is the most commonly used treatment [77]. In confirmed LTBI, the interval between the onset of chemotherapy and the (re)introduction of TNFα inhibitors or other biologic agents varies from 1 to 9 months [76, 77, 101]. The consequences of failing to diagnose patients with LTBI prior to the antirheumatic therapy are serious, as active TB in the course of ongoing immunosuppression often takes an extrapulmonary and disseminated course, depending on the degree of immunosuppression [102, 103, 104, 105, 106, 107]. The usual therapeutic regimen is based on isoniazid, rifampicin, ethambutol, and pyrazinamide [104, 108]. Given the growing resistance of *Mycobacterium*, novel antimicrobials are still urgently needed [109]. Based on the existing reports, early re‐initiation of TNF blockers seems to be both safe and beneficial if clinically indicated and supported by appropriate anti‐TB treatment [110]. However, further research is advisable [111].

The disease activity needs to be determined by laboratory testing at the end or during the TB treatment [78], especially in patients with ILD with new documented lesions of uncertain etiology. The use of isoniazid, rifampicin, or ethambutol can cause several adverse effects, including a rare complication in the form of drug‐induced ILD in about 2% of patients [112, 113, 114]. Reported in such cases, computed tomography findings included bilateral ground‐glass opacities, abnormal lung density, and interstitial infiltrate or thickening [115]. In a few patients diagnosed with isoniazid‐induced pneumonitis, discontinuation of the causative drug and GCS administration proved sufficient to see the episode resolution [115]. It is worth mentioning that pulmonary fibrosis induced by anti‐TB therapy is currently considered extremely rare [115]. Additionally, there is no available data regarding AEs of the pre‐existing ILD during the anti‐TB treatment.

## 8 Summary

**FIGURE 4:**
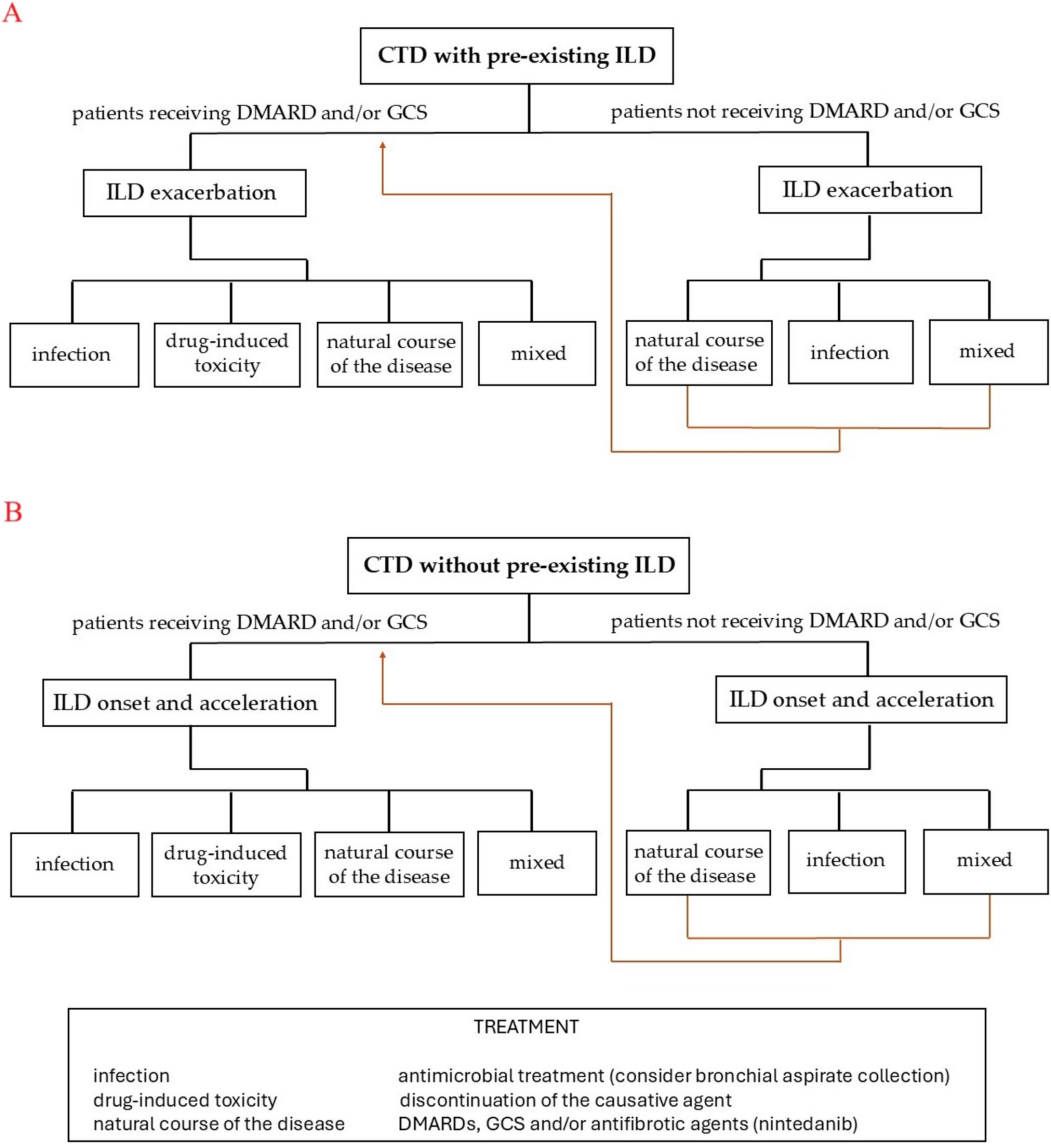
Differential diagnosis of respiratory decompensation in patients with CTD with (A) and without (B) concomitant ILD. Abbreviations: CTD connective tissue disease, ILD interstitial lung disease, DMARD disease‐modifying antirheumatic drugs, GCS glucocorticosteroids.

Acute exacerbation of CTD‐associated ILD, drug‐induced pulmonary injury, nonspecific infections, and tuberculosis can present similar clinical manifestations in the form of fever, dyspnea, and progression of lung infiltrations. It is a challenge to distinguish pulmonary abnormalities associated with the above conditions, given the diversity of radiological patterns and often the lack of their traceability. Multiple factors have to be acknowledged and considered in this group of patients in order to increase the probability of adequate diagnosis and treatment (Figure 4).

Interdisciplinary approach including both pulmonologists and rheumatologists seems necessary. However, the extent of participation of different specialties is not yet clearly defined, and often depends on their availability and cooperation. With an uncertain diagnosis of AE, it is important to consider what will benefit the patient more—intensification of immunosuppression or antimicrobial treatment. Although the clinical dilemma may not always be solvable, there is room for further research. Both the risk of ILD progression and exacerbation have to be investigated in each CTD, including a detailed evaluation of antirheumatic immunosuppressives in terms of a probable drug‐induced injury. Although the available guidelines provide information about long‐term treatment strategies in CTD‐associated ILDs, the management of AEs is still not established. Finally, the processes of TB development in CTD‐associated ILDs should be further investigated.

## Author Contributions

Agata Anna Lewandowska and Cezary Rybacki conceived the concept of the study. Agata Anna Lewandowska and Dorota Waśniowska conducted the analysis and wrote the paper. Cezary Rybacki, Helena Mirus‐Arabik, and Michał Graczyk prepared the presented figures and tables. Ola Duszyńska, Małgorzata Kołodziej, Michał Graczyk, and Aleksandra Gaczkowska collected data.

## Disclosure

The review was conducted using literature available in PubMed, Scopus, and Web of Science medical databases. A thematic analysis was based on the selected articles listed in the references section (years 1986–2025).

## Ethics Statement

The authors have nothing to report.

## Conflicts of Interest

The authors declare no conflicts of interest.

## Data Availability

Data sharing not applicable to this article as no datasets were generated or analysed during the current study.
